# Pulmonary artery agenesis associated with coronary collaterals among adults

**DOI:** 10.1186/s13019-016-0504-1

**Published:** 2016-07-16

**Authors:** Ahmad K. Darwazah, Imad A. Alhaddad

**Affiliations:** Department of Cardiac Surgery, Ramallah and Makassed Hospital, Mount of Olives, Postal Code 91194 Jerusalem, Israel; Department of Cardiology, Jordan Hospital, Amman, Jordan

**Keywords:** Pulmonary artery agenesis, Congenital heart disease, Coronary artery collaterals, Systemic collaterals

## Abstract

Unilateral agenesis of the pulmonary artery is a rare congenital anomaly, which commonly involves the right side. Cases are associated with systemic collaterals, that may also rarely arise from the coronary arteries.

Two adult patients are presented with a right pulmonary artery agenesis associated with collaterals from the right coronary artery. The implications of such an anomaly on pulmonary artery pressure and lung pathology differs among both cases. The association of coronary collaterals is rare and its implication is variable among various patients.

## Background

Unilateral pulmonary artery agenesis is a rare anomaly with variable clinical presentations, it is associated with systemic collaterals together with other cardiovascular anomalies.

In the majority of cases, it is diagnosed and treated early. Rarely, it remains asymptomatic or presents in adults with chest infection, hemoptysis, chest pain, pleural effusion, pulmonary hypertension and congestive heart failure [1, 2].

## Case presentation

### Case one

A 48-year-old male was admitted for evaluation of exertional dyspnea associated with cyanosis.

Since childhood the patient was unable to participate in school activities due to shortness of breath. At the age of 35, he was diagnosed with pulmonary hypertension and received nifedipine, diuretic, warfarin and sildenafil. His condition remained stable till few months before his recent admission.

On admission, he was dyspneic, with oxygen saturation of 75 %. Blood pressure was normal with a regular pulse of 90/min.

There was evidence of central and peripheral cyanosis, clubbing of fingers, elevated jugular venous pressure and bilateral reduction of air entry with scattered fine crepitations. Cardiac examination revealed a right ventricular impulse, a pulmonary ejection click with splitting of the second heart sound. Both limbs were cold, cyanosed and oedematous, the liver was enlarged and haemoglobin was 17 g/dL.

ECG showed right ventricular hypertrophy and right axis with strain pattern. Chest roentgenography showed reduction of right lung volume and vascularity while the left lung was hyperinflated with prominent left pulmonary artery.

Echocardiography showed an enlarged right ventricle and atrium. The pulmonary trunk, left pulmonary artery and proximal part of right pulmonary artery were dilated. Pulmonary pressure was 100 mmHg. Persistent patent ductus arteriosus (PDA) with right to left shunt was seen suggesting Eisenmenger syndrome. The left ventricular size was normal with EF of 45 %.

**Fig. 1:**
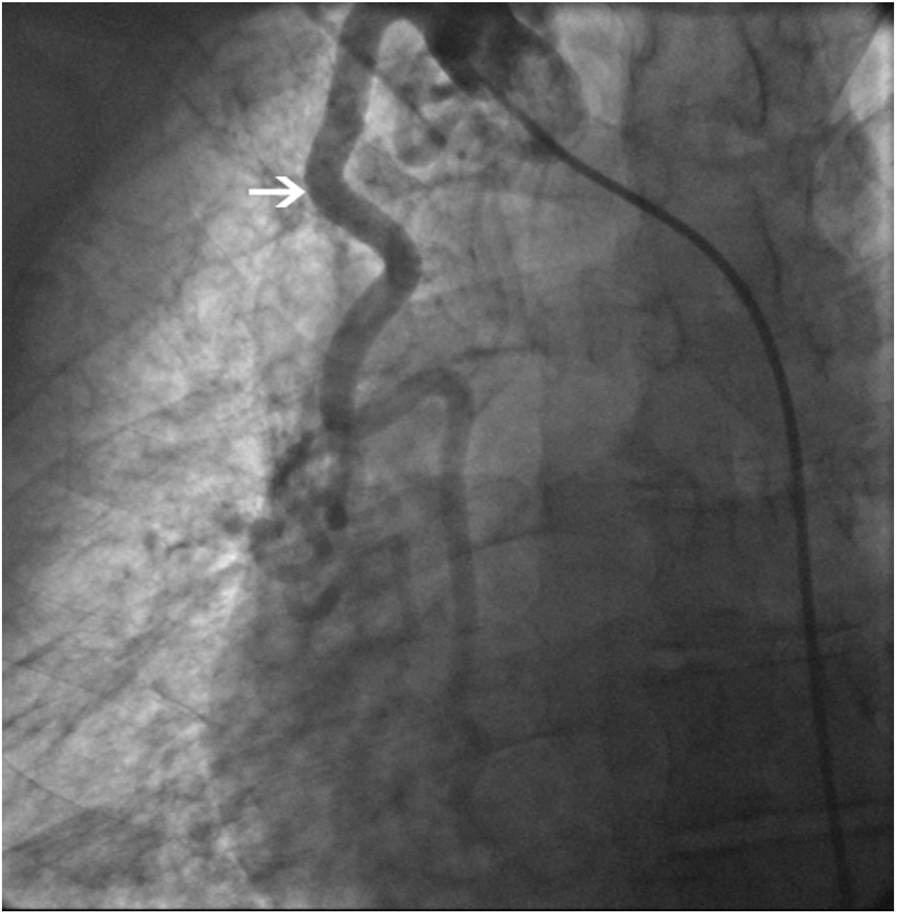
Collaterals from right internal mammary artery (*arrow*)

Coronary angiography revealed an abnormal branch from right coronary artery extending to the right lung with evidence of collaterals from intercostals, paravertebral and right internal mammary artery (Fig. 1).

**Fig. 2:**
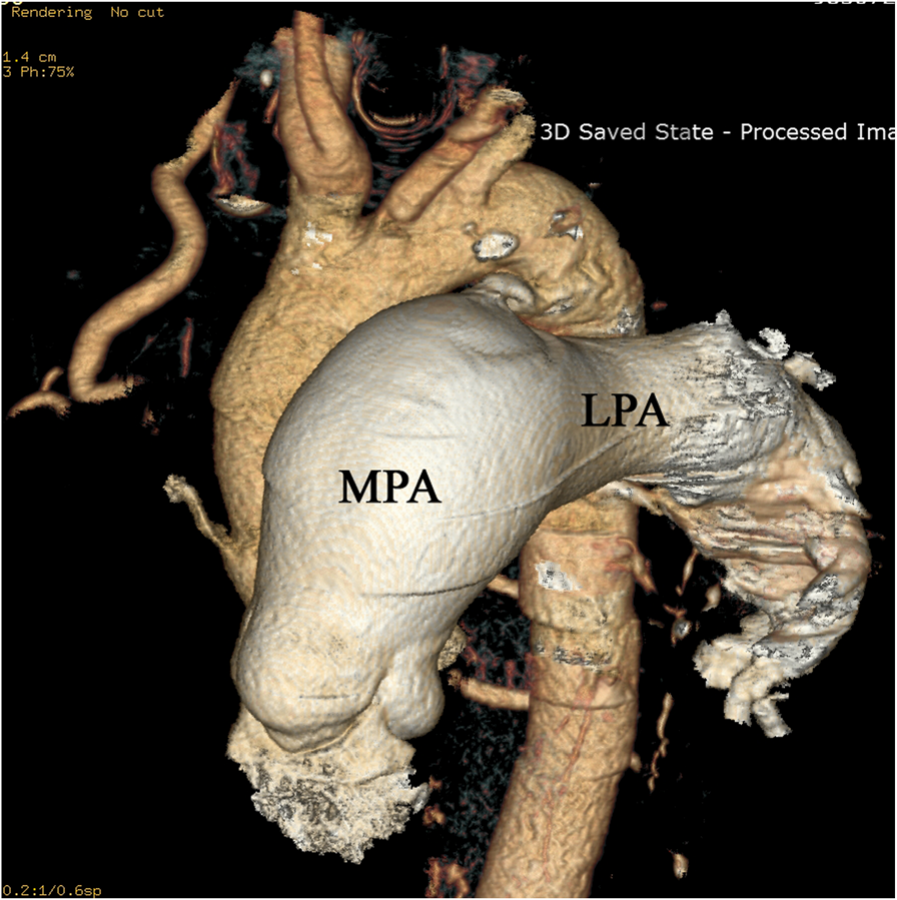
CT angiography showing agenesis of right pulmonary artery. Aneurysmal dilatation of main pulmonary artery (MPA) and left pulmonary artery (LPA) and evidence of PDA

**Fig. 3:**
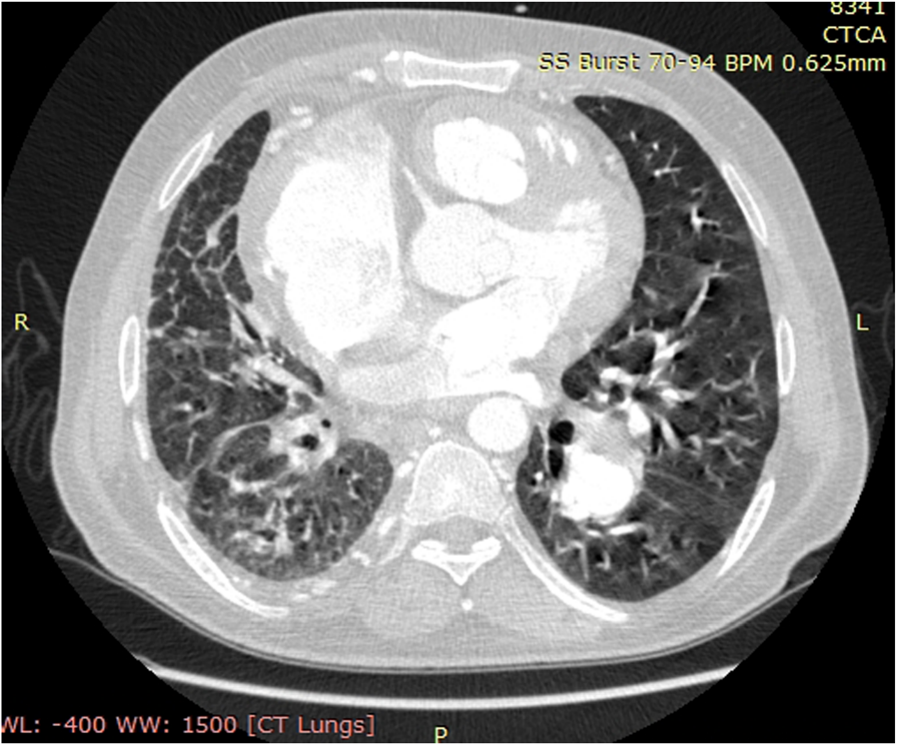
CT chest showing bilateral bronchiectatic changes

CT-angiography revealed agenesis of the main right pulmonary artery (Fig. 2). The right lung was small with cystic bronchiectatic changes and slight changes were seen in the left lower lobe (Fig. 3).

The patient was treated in intensive care with anti failure medications, sildenafil, antibiotics and anticoagulants. He responded well, mean pulmonary artery pressure dropped to 65 mmHg and O2 saturation improved to 90 %.

### Case two

A 58-year-old hypertensive, smoker male was admitted with exertional dyspnea, associated with productive cough alternating with blood tinged sputum.

**Fig. 4:**
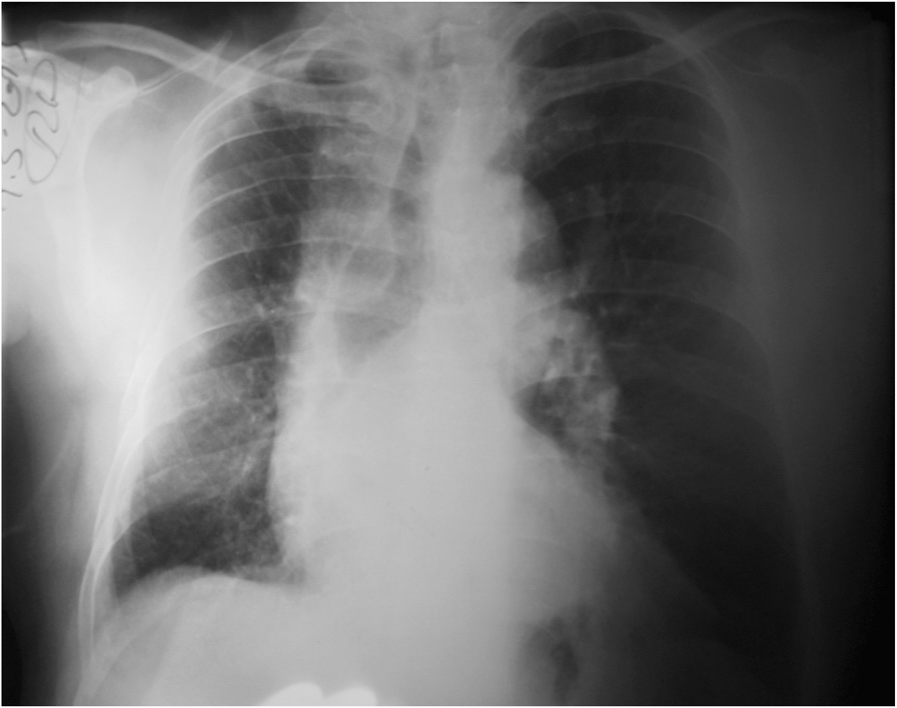
Chest x-ray showing prominent left pulmonary artery with congested hyperinflated left lung and shifting of the mediastinum to the right side

On admission, he looked well but slightly tachypneic. O_2_ saturation was 90 %. Air entry was reduced associated with wheezes and coarse crepitations along right lower lung base. Heart examination was normal. No peripheral oedema, clubbing or cyanosis. Chest roentgenography showed prominent left pulmonary artery with congested hyperinflated left lung and shifting of the mediastinum to the right side (Fig. 4). The right lung was small with slight elevation of the right hemidiaphragm.

**Fig. 5:**
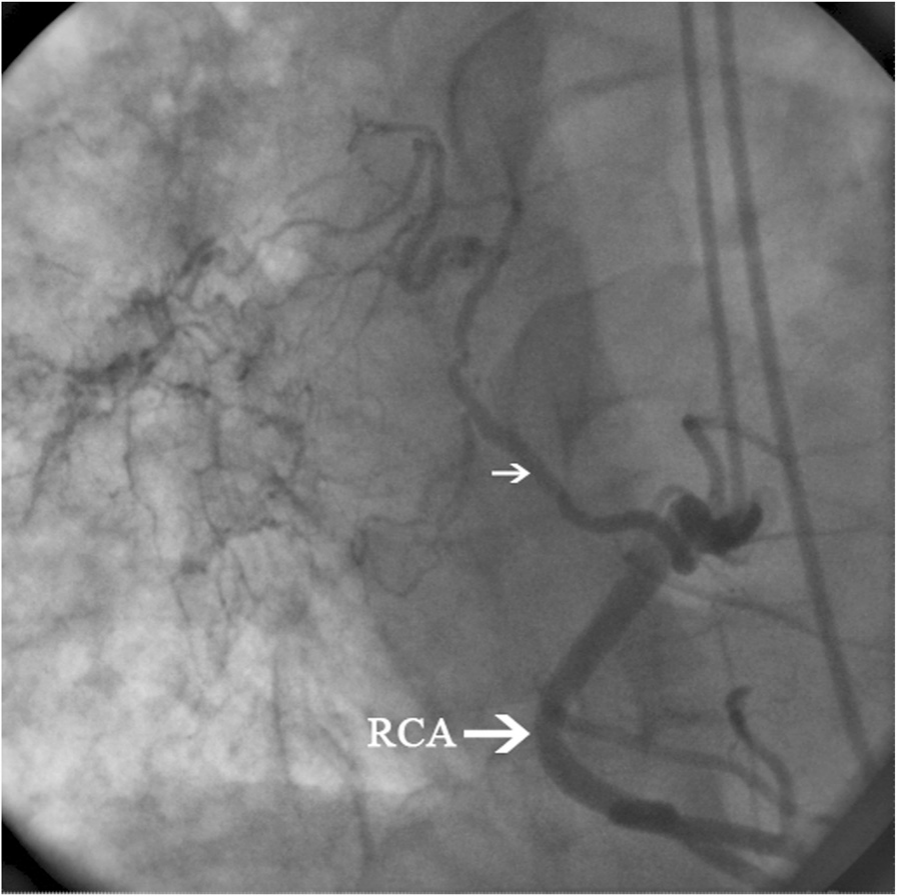
Coronary angiography showing a collateral branch (*small arrow*) from the right coronary artery (RCA) to the hilum of the right lung

Electrocardiography was normal. Echocardiography showed dilated pulmonary artery with mean pressure of 28 mmHg. Coronary angiography showed an abnormal branch extending from the right coronary artery to the hilum of the right lung (Fig. 5). CT angiography revealed complete absence of the right PA with hypoplastic right lung and bronchiectatic changes. Thallium stress test showed no evidence of ischemic changes.

The patient was treated with antibiotics, bronchodilators and calcium channel blockers.

## Discussion

Unilateral agenesis of the pulmonary artery is a rare congenital anomaly with an estimated incidence of 1:200,000 individuals [1].

This anomaly results from involution of the proximal portion of the sixth aortic arch causing absence of the proximal pulmonary artery while, the distal portion is often present as a small vessel or fibrous cord [3]. The intrapulmonary arterial system is present and receives its blood supply from systemic collaterals [4]. Rarely, anomalous collaterals may arise from right or left coronary arteries [2, 4–15].

Agenesis of the right pulmonary artery is more commonly involved [16]. It may occur as an isolated lesion, but in the majority of cases is associated with cardiovascular anomalies [16, 17].

Clinical presentations of pulmonary artery agenesis are variable. The majority of patients are presented with recurrent pulmonary infection, decrease exercise tolerance and dyspnea during exertion. In 20 % of cases, hemoptysis and signs of pulmonary hypertension are seen [16]. 13-30 % of patients may remain asymptomatic leading a benign course and present in adulthood [1, 16].

The diagnosis of PA agenesis is difficult as most patients present with non-specific symptoms. Different diagnostic modalities are often required to reach final diagnosis. Variable findings in the form of absent hilar shadow, decrease pulmonary vascular markings, small hemithorax and ipsilateral cardiac and mediastinal displacement with elevated hemidiaphragm can be seen on chest rentgenography [18].

The use of echocardiography is valuable in diagnosing pulmonary hypertension and associated congenital heart lesions [1]. MRI and high resolution CT scanning are effective modalities in visualisation of the distal pulmonary arteries, bronchiectasis and associated congenital heart disease [1, 19].

The diagnosis of PA agenesis in our patients was delayed for years. The early non-specific symptoms, lack of medical consultation, failure of proper evaluation of chest x-ray and echocardiography and lack of suspicion about the possibility of such a pathology were among the various reasons for such a delay.

The implications of PA agenesis on hemodynamics and lung involvement were completely different among both patients. Failure of closure of ductus arteriosus together with extensive systemic collaterals as seen in the first patient had double edge on hemodynamics, it provided blood supply to the affected side, but at the same time it caused excessive pulmonary blood flow which in turn caused shear stress and intimal damage with the release of vasoconstrictor agents. As a result, the patient presented with severe pulmonary hypertension, reversal of shunt across ductus arteriosus, cyanosis and heart failure.

A different scenario was seen in the second patient, who had no ductus arteriosus nor systemic collaterals. He was presented with stable condition with no pulmonary hypertension, cyanosis or heart failure, also lung involvement in the form of bilateral bronchiectatic changes was extensive in the first patient in comparison to mild unilateral changes in the second case.

The pathogenesis of bronchiectasis is not well established [1]. The difference in lung involvement in our patients could be explained by the degree of impairment of the local lung defence mechanisms and the degree of pulmonary hypertension.

The association between PA agenesis and coronary collaterals is very rare. On reviewing the literature, only thirteen cases were reported (Table 1). The majority of patients were associated with right pulmonary artery agenesis similar to our patients. Collaterals arose either from the right or circumflex coronary artery or both.

**Table 1:** Pulmonary artery agenesis associated with coronary collaterals among adults

Author/year	Age/gender	Site of PA agenesis	Coronary collaterals	Effect on coronary circulation
Thompson JA et al. (1986) [5]	34/M	Left PA	RCA	No effect
Mahnken AH et al. (2000) [6]	49/F	Left PA	CX	No effect
Gupta K et al. (2001)^a^ [7]	64/M	Right PA	CX/RCA	No effect
Kochiadakis GE et al. (2002)^a^ [8]	62/F	Left PA	CX	No effect
Park DY et al. (2003) [9]	55/–	Left PA	CX	Not ruled out
Herper and Korkmaz (2007) [2]	34/M	Right PA	CX/RCA	No effect
Kadi H et al. (2007)^a^ [10]	65/F	Right PA	CX/RCA	Myocardial ischemia
Tseng WC et al. (2010)^a^ [11]	36/–	Right PA	RCA	No effect
De Dominicis F et al. (2011) [4]	44/F	Right PA	RCA	Myocardial ischemia
Soliman A et al. (2012) [12]	27/M	Right PA	CX/RCA	Myocardial ischemia
Nakwan N (2014)^a^ [13]	40/M	Right PA	CX	Myocardial Infarction
Mohan V etal (2014) [14]	46/F	Right PA	RCA	No effect
Mikaberidze N etal (2014)^a^ [15]	71/M	Right PA	RCA	Not ruled out

*PA* pulmonary artery, *M* male, *F* female, *RCA* right coronary artery, *CX* circumflex coronary artery

^a^cases associated with coronary collaterals only

On reviewing these cases, we found that 46 % had collaterals only from coronary arteries. While, the rest had associated systemic collaterals. The implication of associated coronary collaterals is variable. It may impair myocardial perfusion via steal phenomena causing myocardial ischemia [4, 10, 12], infarction [13] or it may have no effect on coronary circulation [7, 8] as the blood flow across the collaterals occurs during systole rather than during diastolic phase [8].

The effect of associated coronary collaterals on myocardial perfusion in our first patient was not evaluated due to his critical condition. However, the patient had no chest pain or electro cardiographic changes denoting myocardial ischemia. Our second patient had no evidence of ischemic changes as proved by thallium scanning.

To date, there is no consensus regarding treatment of PA agenesis. Different surgical and medical modalities of treatment have been used depending upon the age and clinical presentation of these patients.

Early surgical intervention during neonatal period and infancy to restore continuity between main and hilar pulmonary arteries can prevent both morbidity and mortality [17].

Various surgical revascularisation techniques using saphenous vein graft, prosthetic conduit, autologus pericardium, end to end direct anastomosis and reconstruction of “neo” pulmonary artery using ligamentum arteriosus and homograft patch have been used successfully [17, 20]. These procedures may lead to restoration of pulmonary circulation, regression of pulmonary hypertension and development of normal distal pulmonary vasculature [3, 17].

Patients presenting with massive hemoptysis and recurrent pulmonary infection can be treated successfully by either lobectomy, or pneumonectomy with selective embolization of systemic collaterals [21].

Asymptomatic adult patients should be followed up regularly by echocardiography for development of pulmonary hypertension [22]. These patients and those in whom revascularization cannot be performed can be treated successfully by long term vasodilator therapy including calcium channel blockers, prostacyclin infusion, endothelin receptor antagonists and phosphodiesterase inhibitors [16, 23, 24].

Management of the present cases was done medically as the use of surgical revascularization was not applicable. The main target of therapy was to treat respiratory infections and to control pulmonary hypertension by using vasodilator therapy including calcium channel blockers and sildenafil.

Previous studies found that both drugs are effective in reducing pulmonary artery pressure, pulmonary vascular resistance and regression of right ventricular hypertrophy [24–26]. The combination of both medications was effective in controlling pulmonary hypertension with improvement of functional capacity for almost 13 years in our first patient, nevertheless, the patient developed irreversible pulmonary vascular damage. The patient is now under trial of endothelin receptor antagonist (Bosentan) hoping to control pulmonary hypertension until heart lung transplantation becomes available.

Our second patient was given calcium channel blocker as a monotherapy and was kept under observation and follow up by echocardiography.

## Conclusions

Adult patients with PA agenesis have variable presentations and hemodynamic conditions. The presence of PDA and extensive systemic collaterals play a major role in hemodynamics. The association of coronary collaterals is rare and its implication is variable among various patients.

## References

[1] Bouros D, Pare P, Panagou P, Tsinitiris K, Siafakas N (1995). The varied manifestation of pulmonary artery agenesis in adulthood. Chest.

[2] Heper G, Korkmaz ME (2007). High-pressure pulmonary artery aneurysm and unilateral pulmonary artery agenesis in an adult. Tex Heart Inst J.

[3] Toews HW, Pappas G (1983). Surgical management of absent right pulmonary artery with associated pulmonary hypertension. Chest.

[4] De Dominicis F, Leborgne L, Raymond A (2011). Right pulmonary artery agenesis and coronary-to-bronchial artery aneurysm. Interact Cardiovasc Thorac Surg.

[5] Thompson JA, Lewis SA, Mauck HP (1986). Absence of the left pulmonary artery: anomalous collateral from the coronary artery to affected lung. Am Heart J.

[6] Mahnken AH, Wildberger JE, Spüntrup E, Hübner D (2000). Unilateral absence of the left pulmonary artery associated with coronary – to – bronchial artery anastomosis. J Thorac Imaging.

[7] Gupta K, Livesay JJ, Lufschanowski R (2001). Absent right pulmonary artery with coronary collaterals supplying the affected lung. Circulation.

[8] Kochiadakis GE, Chrysostomakis SI, Igoumenidis NE, Skalidis EI, Vardas PE (2002). Anomalous collateral from the coronary artery to the affected lung in a case of congenital absence of the left pulmonary artery. Chest.

[9] Park DY, Lee NH, Choi SH, Yun IS, Park KH, Jung CS, Ko JS, Nam DI, Kim DS, Choi CH, Han KW (2003). Left pulmonary artery agenesis accompanied with fistula of left circumflex artery to left bronchial artery. Korean Circ J.

[10] Kadi H, Kurtoglu N, Karadag B (2007). Congenital absence of the right pulmonary artery with coronary collaterals supplying the affected lung: effect on coronary perfusion. Cardiology.

[11] Tseng WC, Chen YS, Chiu SN (2010). Coronary artery fistula as major source of right lung circulation in a patient with isolated right pulmonary artery agenesis. Eur Heart J.

[12] Soliman A, Jelani A, Eid A, Al Qaseer M. Myocardial Infarction due to Coronary Steal Caused by a Congenital Unilateral Absence of the right pulmonary artery: a Rare Case. BMJ Case Rep. 2012; 8: doi: 10.1136/bcr.04.2011.4108. 10.1136/bcr.04.2011.4108 PMC3316819 22605586

[13] Nakwan N. Congenital unilateral pulmonary atresia with coronary-to-pulmonary collateral artery originating from left circumflex coronary artery. Eur J CardioThorac Surg 2014;1–3:doi:10.1093/ejcts/ezu223. 10.1093/ejcts/ezu223 24872474

[14] Mohan V, Mohan B, Tondon R, Kumbkarni S, Chhabra ST, Aslam N, Wander GS (2014). Case report of isolated congenital absence of right pulmonary artery with collaterals from coronary circulation. Indian Heart J.

[15] Mikaberidze N, Goldberg Y, Khosraviani K, Taub C (2014). Incidentally detected right pulmonary artery agenesis with right coronary artery collateralization. Interact Cardiovasc Thorac Surg.

[16] Ten Harkel AD, Blom NA, Ottenkamp J (2002). Isolated unilateral absence of a pulmonary artery. A case report and review of the literature. Chest.

[17] Welch K, Hanley F, Johnston T, Cailes C, Shah MJ (2005). Isolated unilateral absence of right proximal pulmonary artery: surgical repair and follow-up. Ann Thorac Surg.

[18] Catala FJ, Marti-Bonmati L, Morales-Marin P (1993). Proximal absence of the right pulmonary artery in the adult: computed tomography and magnetic resonance findings. J Thorac Imaging.

[19] Rebergen SA, de Roos A (2000). Congenital heart disease; evaluation of anatomy and function by MRI. Herz.

[20] Green G, Reppert E, Cohlan S, Spencer F (1968). Surgical correction of absence of proximal segment of left pulmonary artery. Circulation.

[21] Rene M, Sans J, Dominguez J, Sancho C, Valldeperas J (1995). Unilateral pulmonary artery agenesis presenting with hemoptysis: treatment by embolization of systemic collaterals. Cardiovasc Intervent Radiol.

[22] Reading DW, Oza U (2012). Unilateral absence of a pulmonary artery: a rare disorder with variable presentation. Proceedings (Baylor Univ Med Center).

[23] Rosenzweig EB, Kerstein D, Barst RJ (1999). Long-term prostacyclin for pulmonary hypertension with associated congenital heart defects. Circulation.

[24] Rodriquez-Gómez F, Martin I, Sànchez A, Pujol E (2006). Sildenafil treatment of unilateral pulmonary edema and pulmonary hypertension in pulmonary artery agenesis. Rev Esp Cardiol.

[25] Ghofrani HA, Schermuly RT, Rose F, Wiedemann R, Kohstall MG, Kreckel A, Olschewski H, Weissmann N, Enke B, Ghofrani S, Seeger W, Grimminger F (2003). Sildenafil for long-term treatment of nonoperable chronic thromboembolic pulmonary hypertension. Am J Respir Crit Care Med.

[26] Ramani GV, Park MH (2010). Update on the clinical utility of Sildenafil in the treatment of pulmonary arterial hypertension. Drug Des Devel Ther.

